# Out of Hand, Out of Control—Fibrous Hamartoma of Infancy of the Hand: A Case Report

**DOI:** 10.1155/crpe/6333096

**Published:** 2026-02-03

**Authors:** Josh Nathan L. Ngai, Agnes R. Mendoza, Mary Beth F. Tanco

**Affiliations:** ^1^ Institute of Pediatrics and Child Health, St. Luke’s Medical Center, Quezon City, Philippines, stluke.com.ph

**Keywords:** case report, fibrous hamartoma of infancy, pediatric hand tumor, Philippines, soft tissue mass

## Abstract

**Background:**

Fibrous hamartoma of infancy (FHI) is a rare benign tumor typically found in the axilla or trunk. Distal extremity involvement is exceptional. We present the first documented case of FHI of the hand in the Philippine literature, highlighting the conflict between oncologic clearance and functional preservation.

**Case Presentation:**

A 7‐month‐old male presented with an enlarging hypothenar mass. Initial ultrasonography revealed a heterogenous mass with intralesional cystic spaces, leading to a misdiagnosis of hemangioma despite the absence of significant flow on Doppler interrogation. Propranolol treatment failed. MRI revealed a poorly encapsulated mass encasing muscles, raising suspicion for malignancy. Intraoperatively, the tumor appeared as matted tissue with nondelineated borders, encroaching on neurovascular structures. To preserve hand function, a subtotal excision was performed rather than radical en bloc removal. Histopathology confirmed the triphasic components diagnostic of FHI.

**Conclusion:**

Local recurrence was noted 6 months postoperatively before the patient was lost to follow‐up. This case underscores the diagnostic challenge of FHI in the hand due to vascular mimicry. Furthermore, the tumor’s infiltrative nature in complex anatomy forces a difficult trade‐off: Radical excision offers cure but risks functional devastation, whereas functional preservation carries a high risk of recurrence.

## 1. Introduction

Since first being described by Reye in 1956, the global index of fibrous hamartoma of infancy (FHI) has grown significantly. While early reviews cited approximately 200 cases, recent large‐scale clinicopathologic studies have expanded the count to over 450 [1–3]. FHIs are rare, benign, subcutaneous fibrous proliferations that have unique clinicopathologic features—comprised of triphasic pattern of mature fibrocollagenous tissue, mature adipose tissue, and primitive mesenchymal cells. Typically presenting as a rapidly growing solitary mass‐like lesion in the subcutaneous layer, they occur in the first 2 years of life and are primarily located on the upper limb girdle [4, 5]. While involvement of other sites has been documented, distal extremity involvement remains exceptionally rare, with fewer than 15 detailed reports in the English literature explicitly describing FHI isolated to the hand [6–13]. The treatment of choice is local excision, and even with incomplete excision, recurrence rate is low and prognosis remains excellent.

This report describes a rare case of FHI in the hand misdiagnosed as a vascular anomaly, aiming to discuss the specific surgical dilemma where radical excision must be weighed against functional morbidity.

This manuscript was prepared following the CARE guidelines (https://www.care-statement.org).

## 2. Case Report

The patient is a 7‐month‐old male presenting with a progressively enlarging, painless, and solitary mass on the lateral aspect of his right hand encompassing the dorsum and palmar aspect. The patient was born to a primigravid, at full term via normal vaginal delivery, and was noted to not have any feto‐maternal complications or congenital anomalies. The patient was reportedly well and had normal growth and development up until 2 months of age, when a firm, nonmovable, well circumscribed mass, measuring approximately 1‐2 cm, was noted on the hypothenar eminence of the patient’s right hand. There were no other associated signs and symptoms with note of intact motor function and sensation of the affected hand.

Ultrasonography of the right hand was performed revealing an ill‐defined, heterogeneously iso‐ to slightly hyperechoic soft tissue mass surrounding the 3^rd^ to 5^th^ metacarpals. Few intralesional cystic spaces were also noted within the mass. Crucially, color Doppler interrogation was performed and did not demonstrate significant internal vascularity. Based on these morphological features, the lesion was interpreted as a benign soft tissue vascular malformation such as a hemangioma, despite the lack of documented high‐velocity flow on Doppler interrogation. On that impression, the patient was advised for regular follow‐ups for monitoring of size of mass on the hope that it may spontaneously regress/involute.

During the interim however, mass was noted to have progressively increased in size. On an unrelated consult due to fall injury, the patient underwent repeat ultrasonography of the hand, at 5 months of age, which again noted similar findings but with increase in size estimated at ∼2.3 × 3.1 × 3.4 cm in its palmar aspect and ∼2.3 × 3.1 × 3.1 cm in its dorsal aspect, still under the consideration of a soft tissue vascular malformation (i.e., hemangioma). The patient was referred to a pediatric cardiologist and was started on propranolol in hopes of decreasing the size and even allowing for complete resolution of the suspected vascular mass.

**Figure 1:**
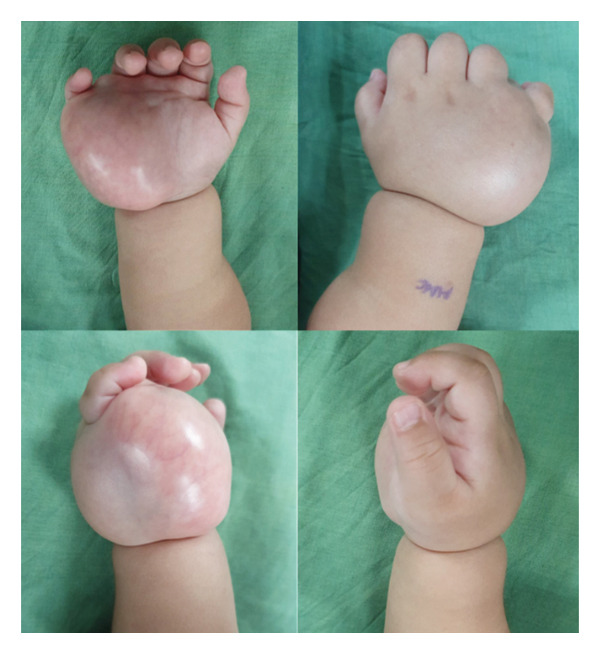
Appearance of the right hand prior to excision. Note the extent of the mass involving the entirety of the dorsum and palmar aspects of the hypothenar area of the right hand. There is also mass effect deforming the interdigital distance between the 4^th^ and 5^th^ digits. Telangiectasia can also be appreciated on the palmer surface of the skin overlying the mass.

After 1 month of intake of propranolol, there was still noted progressive increase in size of the mass. Consult was sought, and mass was noted to now measure roughly ∼5 × 5.4 cm on examination, still firm and not movable, with associated distortion of the interdigital distance between the 4^th^ and 5^th^ fingers, but with preservation of motor and sensory function (Figure 1). On the assessment of other possible soft tissue tumors, propranolol was discontinued and the patient was advised to undergo MRI under sedation for better characterization and delineation of the mass for possible surgical biopsy or excision down the line.

**Figure 2:**
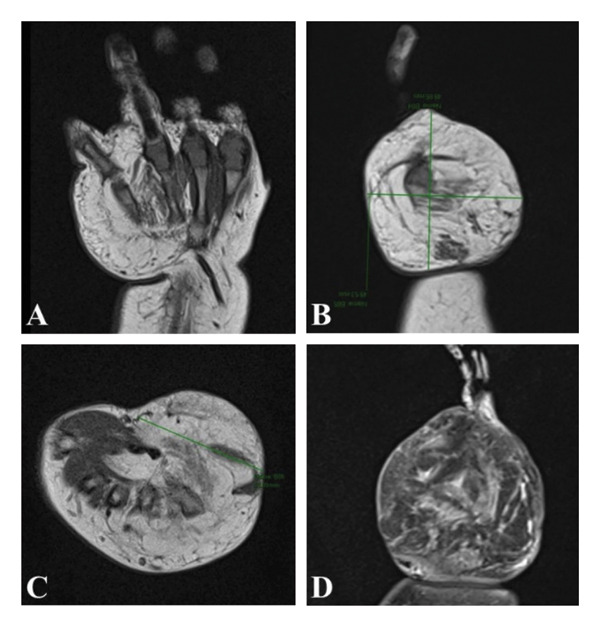
Magnetic resonance imaging of the right‐hand mass. (A–C) Note the enlarged mass measuring approximately 4.95 × 3.9 × 4.98 cm (anteroposterior × transverse  × craniocaudal aspect) on the dorsal and palmar aspect of the hypothenar aspect of the right hand encasing the adjacent bony, muscular, and neurovascular structures without grossly deforming the internal architecture and structures. There is appreciable signal enhancement on T1‐weighted imaging revealing a predominantly fatty lesion. (D) There is heterogenous uptake of gadolinium within the mass lesion on T2‐weighted imaging.

MRI findings of the mass revealed an ill‐defined, predominantly fatty mass on the hypothenar region, measuring 4.95 × 3.9 × 4.98 cm, with encasement of hypothenar muscles and 3^rd^ to 5^th^ metacarpal bones, with bowing of the 5^th^ metacarpal, with no abnormal signals of note in the rest of the neurovascular or muscular structures (Figure 2). Considerations drawn from the MRI were hemangioma with fatty involution, lipoma, lipoblastoma, or liposarcoma, with other possible soft tissue tumors.

**Figure 3:**
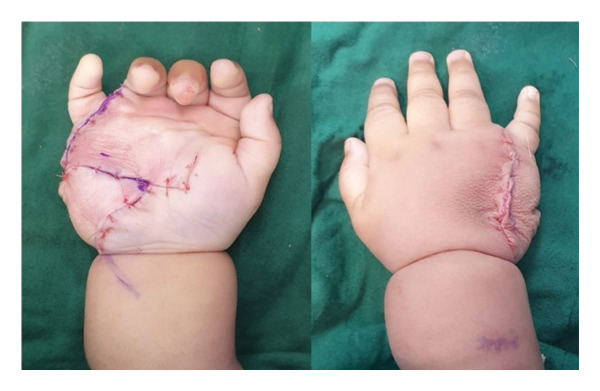
Postexcisional appearance of the right hand with notable significant decrease in hand bulk due to removal of mass.

The patient was referred to an orthopedic surgeon and was advised excision of the right‐hand mass which the patient subsequently underwent. Intraoperatively, the mass presented as matted subcutaneous tissue with nondelineated borders, encroaching significantly on the flexor tendons and neurovascular structures. Given the preoperative consideration of malignancy, a zigzag incision was utilized. However, the tumor’s extensive interdigitation precluded en bloc resection without sacrificing hand function. Consistent with preoperative counseling and shared decision‐making, a strategic decision was made to perform a function‐sparing subtotal excision to preserve neurovascular integrity, rather than a radical resection that would compromise hand function, with the parents explicitly acknowledging the trade‐off of a higher risk of local recurrence (Figure 3).

**Figure 4:**
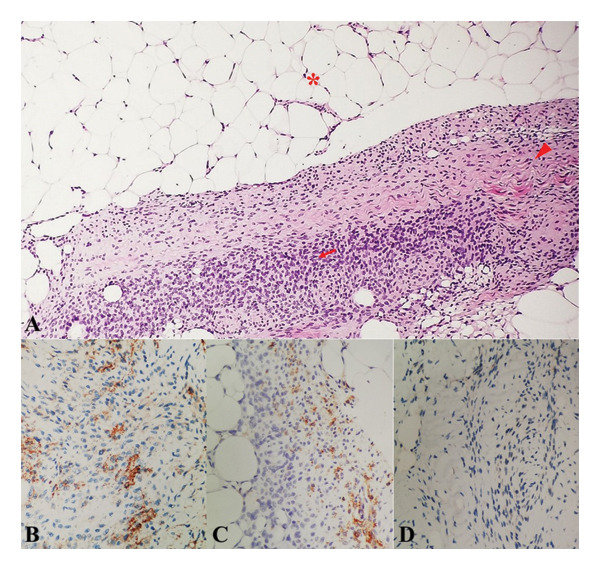
Histologic and immunohistochemical study of hand mass. (A) Fibrous hamartomas of infancy are characterized by an organoid triphasic mixture of 3 distinct components: mature fibrocartilaginous tissue (arrow head), mature adipose tissue (asterisk), and primitive mesenchymal cells (arrow) (scanner view). (B) CD34 staining (brown) focally positive in fibroblastic fascicles (100x magnification). (C) SMA staining (brown) positive in fibroblastic fascicles and negative in primitive mesenchyme component (100x magnification). (D) S‐100 staining negative in spindle cells (100x magnification).

On pathologic evaluation, the mass was noted to be grossly tan and predominantly fibrous in consistency and histologically comprised of three components: mature adipose tissue; spindle cells; and small round blue cells with morphologic features similar to FHI and lipofibromatosis. The sample underwent immunohistochemical staining, noting positivity in SMA, CD34, and S100 which taken together were compatible with FHI, establishing its diagnosis. Specific immunohistochemical staining showed CD34 positivity in the mesenchymal components and SMA positivity in the fibroblastic fascicles. S‐100 protein was positive only in the mature adipose tissue and negative in the spindle and primitive mesenchymal cells, ruling out neural and primary adipocytic tumors (Figure 4).

Following confirmation of the benign diagnosis via immunohistochemical staining, a multidisciplinary discussion was held between the orthopedic surgeon and the attending pediatrician. It was determined that re‐excision to achieve negative margins would carry unacceptable functional morbidity. Therefore, no further surgical intervention was performed. The parents were counseled regarding the benign nature of the lesion and risk of local recurrence, and a plan for observation was agreed upon.

However, the parents reported a slow regrowth of the mass 6 months postoperatively. This recurrence was confirmed by the parents over follow‐up teleconsultation with the patient’s attending pediatrician, where the mass appeared to have regained near‐preoperative bulk. Following this recurrence, the patient was referred for oncologic evaluation but was subsequently lost to follow‐up. Consequently, long‐term functional outcomes could not be assessed.

## 3. Discussion

FHI is a rare benign soft tissue lesion that occurs in the first 2 years of life. The average age of diagnosis is ∼8 months of age. It is reported to have a higher predilection for boys with a male to female ratio of 2.4:1 [5]. Roughly 25% of cases have been attributed congenitally; however, FHI is not known to have any familial or syndromic associations [8]. FHI is commonly located in the upper trunk and groin area, although they may appear in any part of the body with lesser documented sites such as the hands or feet, as presented here. The lesion presents as a poorly demarcated, unencapsulated, firm, painless, solitary nodule measuring as large as > 20 cm, with a size of, on average, < 5 cm [14, 15]. Typically a slowly enlarging lesion, it may be freely movable or fixed to surrounding tissue, such as in this patient, giving cause for concern of possible malignancy. Some have documented disruption of adjacent architecture, specifically the skin with changes that include alteration in pigmentation, eccrine gland hyperplasia, and hypertrichosis [15]. These findings were not seen in this patient. However, telangiectasia was noted over the skin on the palmar aspect of the hand overlying the lesion.

Upon review of literature, to the best of our knowledge, this represents the first documented case of FHI in the Philippines. This case presents the uncommon feature of being exclusively isolated to the hand with support of diagnosis by way of immunohistochemical staining despite having malignant features (i.e., firm, fixed, with encroachment over surrounding neurovasculature and musculature) [7–13].

Diagnosis of FHI can typically be made by way of routine hematoxylin and eosin staining of an adequately taken sample. The tumor is characterized by triphasic components of well‐defined intersecting trabeculae of dense fibrocollagenous tissue, areas of immature‐appearing, small, rounded, primitive mesenchymal cells, and mature adipose tissue, in varying proportions forming organoid structures. The fibrous fascicles resemble fibromatosis. Immature mesenchymal cells can be arranged into different configurations such as small nests, whorls, or bands while mature adipose tissue is typically interspersed between the other structural elements [14]. Anaplasia is not common in this tumor; however, the presence of high cellularity and immature cells may lead to an erroneously diagnosed malignant entity.

In instances where the histopathologic diagnosis is in question, then it is important to consider other soft tissue tumors, benign or malignant, that may present similarly both clinically and histologically. The differential diagnoses that can be considered here are infantile digital fibromatosis, myofibroma, lipofibromatosis, aponeurotic fibroma, histiocytoma, dermatofibroma, leiomyosarcoma, and fibrosarcoma [8, 16]. Histopathologically, lipofibromatosis is the primary differential diagnosis in the distal extremities. However, the distinction lies in the architecture, wherein lipofibromatosis is a biphasic tumor (adipose and fibroblastic), lacking the specific organoid structures of FHI [5]. FHI is definitively distinguished by its triphasic nature, specifically the presence of the third component: discrete nests of primitive, small round cells in a myxoid stroma (the organoid pattern), as seen in our patient. The extensiveness of these clinicopathologic entities only serves to bolster the importance in the use of adjunctive techniques in aiding the correct diagnosis such as through the use of immunohistochemical staining and cytogenetic studies [8, 17]. Fibroblastic component of the lesions is SMA positive. Mesenchymal elements are CD34 positive, partially SMA positive, and S‐100 negative, while mature adipose tissue is S‐100 protein positive [7, 8, 14]. In this case, histologic findings were congruent with the typical triphasic pattern of tissue differentiation seen in FHI. Furthermore, S‐100 positivity was restricted strictly to the mature adipose tissue, ruling out neural tumors. Immunohistochemical staining only served to further reinforce the definitive diagnosis of FHI (Figure 4).

Imaging studies of FHI have not been fully investigated due to the rarity of the lesion; however, their use can provide some insight as to the possible entity afflicting patients with soft tissue tumors. X‐ray imaging has been used and documented although the information it provides is of little value since FHI only demonstrates soft tissue characteristics [4]. Ultrasonography can be a helpful tool and is fundamentally invaluable in its use for the evaluation of palpable lesions, especially in young children. Although not well established, ultrasonography has been shown to reveal distinct signal patterns only appreciable in FHI. It is characteristically visualized as a mass with heterogeneous hyperechogenicity formed by intervening hypoechoic portions in the hyperechoic mass coined as the “serpentine pattern.” Here, the hyperechoic portions represent the fat component and the hypoechoic portions can be the fibrous component of the lesion on pathology. Ultrasonography also does not show appreciable vascularity in this lesion [18, 19].

In this case, although not described as “serpentine,” the US report did describe a pattern of an “ill‐defined, heterogenous, predominantly slightly hyperechoic mass with some intralesional cystic spaces,” leading to the erroneous diagnosis of a vascular malformation. However, the primitive myxoid mesenchymal component can appear anechoic or cystic. In our case, these “cystic spaces” led to the misdiagnosis of hemangioma [18]. In the absence of Doppler flow, these spaces likely represented the primitive myxoid mesenchymal component of the FHI, which can appear hypoechoic and mimic fluid‐filled channels. It is critical to note that FHI is typically avascular or shows minimal flow on color Doppler. This finding is congruent with the findings documented in the literature; however, this is nonspecific, which may have been the reason why a vascular lesion (i.e., hemangioma) was the primary consideration of the mass until MRI was done [18]. Unfamiliarity of this rare lesion may have also played a role in how documentation of the US findings was done [20].

Magnetic resonance imaging is valuable not only for delineating the extent of soft tissue lesions but also for characterizing their histological composition. In FHI, composition is well delineated. When composed primarily of adipose tissue, it will show high signal intensity on T1‐weighted images. If composed primarily of fibrous tissue, then it will show low signal intensity on T1‐weighted images [9, 11, 21]. On T2‐weighted images, the tumor shows mixed intensity with heterogeneous enhancement after gadolinium is given [21]. In this case, the mass was documented as a poorly encapsulated, predominantly fatty lesion exhibiting hyperintense signal on T1‐weighted images, hypointensity on fat‐suppressed sequences, and mixed contrast enhancement (Figure 2). Given these characteristics together with age and location of the lesion, other myofibroblastic and adipocytic tumors can be ruled out. Infantile fibromatosis, myofibromatosis, and congenital fibrosarcomas can be excluded due to the high fat signals. Lipoma and liposarcoma may be excluded as they do not present with signals of fibrous strands and dilated vessels on MRI [4]. Lipofibromatosis however may be difficult to exclude on imaging alone but may be done definitively though histopathology.

The treatment of choice remains to be complete excision with an enveloping margin of normal tissue. Although the clinical course is benign, the lesion may be deeply spread and locally invasive. Radical excision is necessitated in order to avoid local relapses, which, although rare, has been reported to occur in ∼12% to 15% of cases, typically within the first few months after primary surgery [3, 22]. Local recurrence has been attributed to incomplete excision, which may have been done for several reasons but primarily due to its poor circumscription, irregular boundaries, and involvement of local structures, such as seen in this case where the lesion was only partially resected. Minimal residual disease is acceptable given the benign course of the lesion and in cases where complete surgery would result in serious anatomical or functional impairment [3, 22]. Radical excision in the hand carries documented risks of flexion contractures and functional deficits. In cases where total resection would result in mutilation or severe functional loss, conservative management or subtotal excision is a recognized and valid option [7, 21]. Although recurrence rates are rare and prognosis tends to be excellent overall, spontaneous regression has not been documented, thus necessitating the need for close follow‐up. For instances wherein conservative re‐excision is not feasible, it has been documented that a “wait and see” approach is acceptable [22]. In this patient, who continues to be developmentally and functionally well, there had been local relapse of the mass 6 months postexcision, highlighting the difficult trade‐off inherent in managing FHI in complex anatomical sites.

## 4. Conclusion

As the first documented case of FHI of the hand in the Philippine literature, this report establishes a vital regional precedent for recognizing this rare, deceptive tumor that frequently mimics vascular anomalies and leads to significant diagnostic delays. The tumor’s infiltrative growth amidst critical neurovascular structures creates a severe surgical dilemma, forcing a trade‐off between radical excision for oncologic cure and the preservation of essential hand function. Consequently, clinicians must accept that partial excision may be necessary to prevent permanent disability, provided that families are thoroughly counseled regarding the high risk of local recurrence and the absolute necessity of rigorous long‐term surveillance when functional sparing is prioritized.

## Author Contributions

All authors attest that they meet the current ICMJE criteria for authorship.

## Funding

No funding was received for this manuscript.

## Ethics Statement

Written informed consent was obtained from the patient’s parents for the surgical procedure on the day preceding the elective procedure. Verbal informed consent was obtained from the parents for the publication of this case report and the use of clinical photographs, which were voluntarily provided by the parents themselves. The patient’s identity has been anonymized in all images and text.

## Conflicts of Interest

The authors declare no conflicts of interest.

## Data Availability

The data that support the findings of this study are available on request from the corresponding author. The data are not publicly available due to privacy or ethical restrictions.
